# Neutralizing Monoclonal Antibodies against Disparate Epitopes on Ricin Toxin’s Enzymatic Subunit Interfere with Intracellular Toxin Transport

**DOI:** 10.1038/srep22721

**Published:** 2016-03-07

**Authors:** Anastasiya Yermakova, Tove Irene Klokk, Joanne M. O’Hara, Richard Cole, Kirsten Sandvig, Nicholas J. Mantis

**Affiliations:** 1 Division of Infectious Disease, Wadsworth Center, New York State Department of Health, Albany, NY 12208; 2 Department of Biomedical Sciences, School of Public Health, University at Albany, Albany, NY 12201; 3 Department of Molecular Cell Biology, Centre for Cancer Biomedicine, Institute for Cancer Research, The Norwegian Radium Hospital, Oslo University Hospital, Montebello, Oslo, Norway; 4 Department of Biosciences, University of Oslo, Oslo, Norway; 5 Division of Translational Medicine, Wadsworth Center, New York State Department of Health, Albany, NY 12201.

## Abstract

Ricin is a member of the A-B family of bacterial and plant toxins that exploit retrograde trafficking to the Golgi apparatus and endoplasmic reticulum (ER) as a means to deliver their cytotoxic enzymatic subunits into the cytoplasm of mammalian cells. In this study we demonstrate that R70 and SyH7, two well-characterized monoclonal antibodies (mAbs) directed against distinct epitopes on the surface of ricin’s enzymatic subunit (RTA), interfere with toxin transport from the plasma membrane to the trans Golgi network. Toxin-mAb complexes formed on the cell surface delayed ricin’s egress from EEA-1^+^ and Rab7^+^ vesicles and enhanced toxin accumulation in LAMP-1^+^ vesicles, suggesting the complexes were destined for degradation in lysosomes. Three other RTA-specific neutralizing mAbs against different epitopes were similar to R70 and SyH7 in terms of their effects on ricin retrograde transport. We conclude that interference with toxin retrograde transport may be a hallmark of toxin-neutralizing antibodies directed against disparate epitopes on RTA.

Ricin belongs to the A-B family of medically important plant and bacterial protein toxins that exploit retrograde transport through the Golgi apparatus and endoplasmic reticulum (ER) to gain entry into the cytoplasm of host cells^1^,^2^. Ricin’s binding subunit (RTB) is a galactose- and N-acetylgalactosamine (Gal/GalNAc)-specific lectin that facilitates receptor-mediated endocytosis of ricin holotoxin via clathrin-dependent and -independent mechanisms. RTB is also required for trafficking of ricin to the trans-Golgi network (TGN) and ER. Within the ER, ricin’s catalytic subunit (RTA) is liberated from RTB by virtue of protein disulfide isomerase (PDI) and then dislocated into the host cell cytosol via the Sec61 translocon^3^,^4^. RTA is an RNA N-glycosidase that cleaves the N-glycosidic bond of a conserved adenine residue within the sarcin-ricin loop of eukaryotic 28S ribosomal RNA, resulting in protein synthesis arrest and cell death by apoptosis.

We are interested in the underlying mechanisms by which antibodies neutralize ricin, and applying this information to the development of much needed medical countermeasures against the toxin, including a subunit vaccine and immunotherapeutics^5^. Surprisingly, the majority of ricin toxin-neutralizing monoclonal antibodies (mAbs) that have been identified to date are directed against RTA, not RTB. R70 (also known as UNIVAX70/38), for example, is a murine IgG1 mAb directed against a linear epitope within an immunodominant loop-helix-loop motif of RTA known as α-helix B (Supplementary Table 1; Supplementary Fig. 1)^6^,^7^. R70 neutralizes ricin in Vero cell-based assays with an IC_50_ of ~50 ng/mL and passively protects mice against systemic and mucosal toxin challenges^8^. At least four other R70-like mAbs, including PB10, have been described, each with potent toxin-neutralizing activity^9^,^10^. The mAb SyH7 defines a second immunodominant region on RTA (Supplementary Table 1)^10^. SyH7 recognizes a linear epitope spanning residues 187–198 and is equally potent at neutralizing ricin toxin *in vitro* as R70^10^. We recently described three other SyH7-like mAbs, each with the capacity to passively protect mice against ricin toxin challenge^9^.

It remains unclear how RTA-specific mAbs like R70 and SyH7 neutralize ricin. It has been proposed that R70-like antibodies may affect RTA’s RNA N-glycosidase activity through distortion of α-helix B^11^. While there is evidence to suggest R70 marginally impacts RTA’s enzymatic activity in cell free *in vitro* translation assays^8^, it seems unlikely that R70 would ever encounter RTA in the cytoplasm, considering that RTA only reaches its final destination as a consequence of retrograde transport and retro-translocation. Rather, we think it more likely that R70 and SyH7 interfere with an upstream event in the intoxication process. Pincus and colleagues suggested that certain toxin-neutralizing, RTA-specific murine mAbs delay toxin internalization and/or interfere with intracellular trafficking to the ER^12^. We concur with this model and, based on numerous studies from our group, would argue more specifically that ricin RTA-specific mAbs likely influence very upstream events in the retrograde trafficking pathway, ultimately impairing delivery of ricin to the TGN^13^,^14^,^15^,^16^. In the current study we demonstrate using a combination of confocal microscopy and TGN-specific labeling methods that R70 and SyH7, as well as three other toxin-neutralizing RTA-specific mAbs impair retrograde trafficking of ricin to the TGN.

## Results

### Uptake and intracellular trafficking of R70- and SyH7-toxin complexes into adherent cells

**Figure 1:**
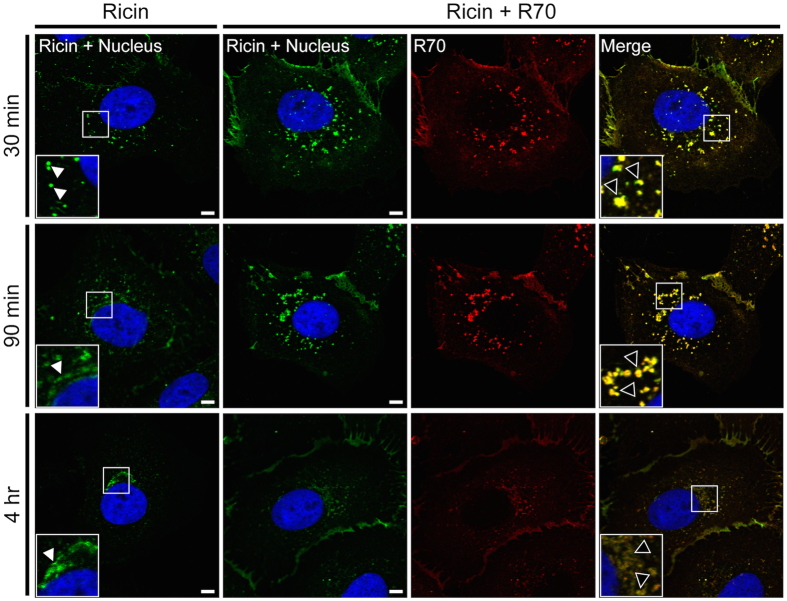
R70 is internalized in complex with ricin and delays toxin accumulation in TGN. Vero cells, grown on glass coverslips, were cooled to 4 °C and incubated for 30 min with ricin-FITC. The cells were then washed, treated (or not) with R70 for an additional 30 min at 4 °C and then shifted to 37 °C. At the indicated time points (30 min, 90 min and 4 hr) the cells were fixed, stained with DyLight^®^ 549-labeled secondary Ab and imaged by confocal microscopy. In the images, ricin appears green, R70 is red, a merge between ricin and R70 is yellow, and the cell nucleus is blue. Insets in the right and left hand columns highlight the subcellular localization of ricin (white arrowheads) and ricin-R70 complexes (black arrowheads). Images are representative of at least 5 independent experiments. Scale bar, 5 μm.

To examine whether R70 and SyH7 are internalized into cells in complex with ricin, Vero cells were grown overnight on glass coverslips and then incubated with FITC-labeled ricin holotoxin for 30 min at 4 °C to allow toxin binding but not endocytosis. The cells were then washed to remove unbound toxin, treated with R70 or SyH7 for additional 30 min at 4 °C and then shifted to 37 °C to permit toxin internalization. At time points thereafter (30 min, 90 min and 4 hr), the cells were fixed, probed with DyLight^®^ 549 anti-mouse IgG and visualized by confocal laser scanning microscopy (CLSM). We observed that R70- and SyH7-toxin complexes were internalized and trafficked intracellularly in Vero cells, as evidenced by colocalized staining of ricin (green) and R70 or SyH7 (red) at each of the three time points examined (Fig. 1; Supplementary Fig. 2). At the 30 min time point, toxin-mAb complexes were situated within vesicles that were distributed throughout the cytoplasm. By 90 min, the toxin-mAb complexes resided within vesicles that had localized around the nucleus. By 4 hr, vesicles containing toxin-mAb complexes had largely dissipated, although residual ricin-mAb staining was still evident around the perinuclear space.

**Figure 2:**
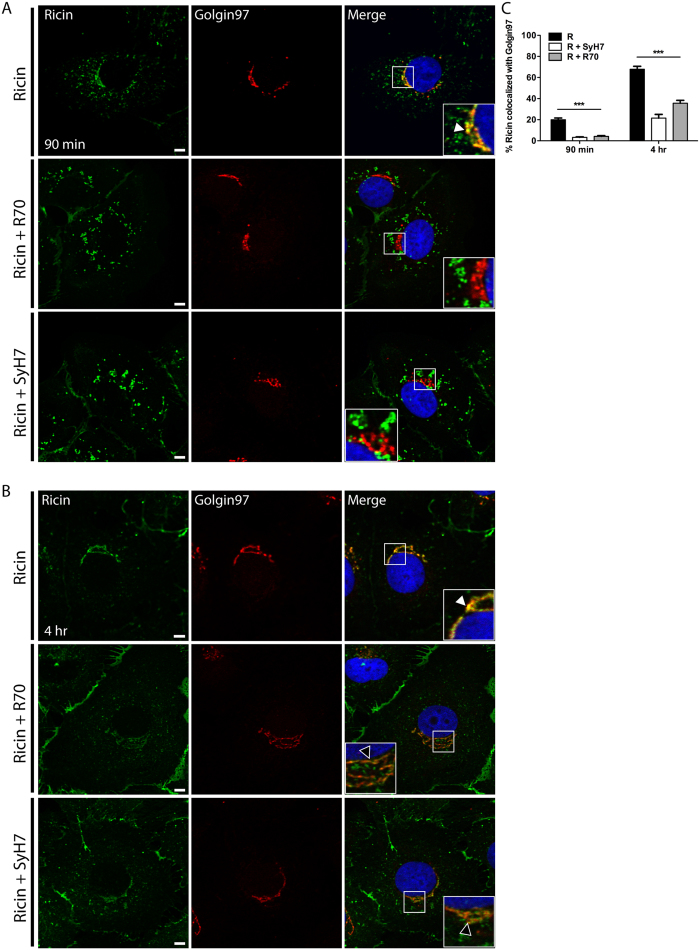
R70 and SyH7 reduce ricin accumulation in the TGN. Vero cells, grown on glass coverslips, were cooled to 4 °C and treated with ricin, ricin-R70 or SyH7-ricin. Cells were collected at 90 min or 4 hr (as described above) and then immunolabeled with Golgin97 to localize the TGN. Representative images taken at the (**A**) 90 min and (**B**) 4 hr time point indicating the co-localization of ricin with Golgin97 in the absence (top panels) but not in the presence of R70 (middle panels) or SyH7 (bottom panels). The arrowhead (inset, top right panels in **A**,**B**) indicates colocalization between ricin (green) and Golgin97 (red) staining. Scale bar, 5 μm. (**C**) The frequency of ricin colocalization with Golgin97 at the indicated time points was quantitated with ImageJ, as described in the Materials and Methods. At least 20–30 cells were analyzed from each time point. ***p < 0.001 determined using a one-way ANOVA with Tukey’s post-test.

To determine whether R70 and SyH7 interfere with transport of ricin to the TGN, Vero cells were treated with mAb-ricin complexes and then subjected to immuno-labeling with Golgin97 (Fig. 2). The trafficking of ricin to the TGN was significantly impaired by R70 and SyH7 (50–60%; p < 0.05) (Fig. 2A–C) but not FGA12, a non-neutralizing RTA-specific isotype control mAb^10^,^15^ (Supplementary Fig. 3). It should be acknowledged that the failure of FGA12 to interfere with intracellular ricin transport is likely due to FGA12’s low affinity for soluble toxin and not necessarily related to epitope specificity per se.

**Figure 3:**
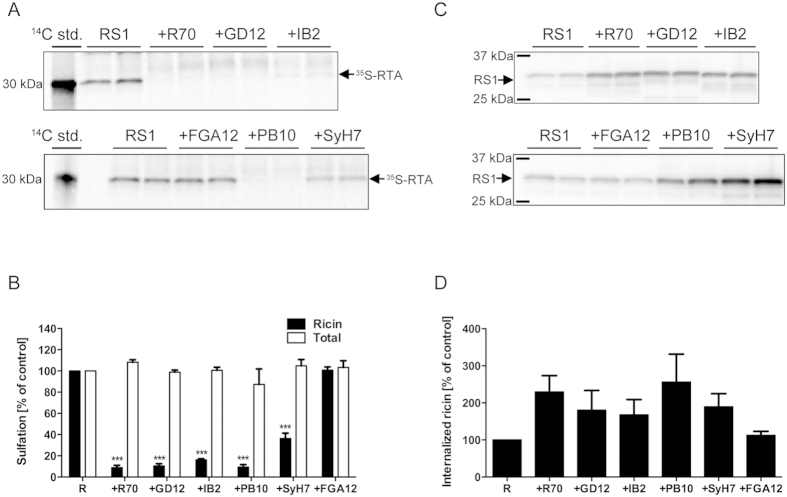
Inhibition of ricin trafficking to the TGN by R70, SyH7 and other toxin-neutralizing, RTA-specific mAbs. HeLa cells were incubated with Na_2_^35^SO_4_ prior to the addition of RS1 in the absence or presence of the indicated mAbs. Two hours later the cells were washed with buffer containing lactose (0.1 M) to remove residual surface-bound ricin and then lysed. Precipitated proteins from lysates, as well as a^14^C-methylated protein molecular weight standard, were subjected to SDS-PAGE and transferred to a PVDF membrane. Specific RTA sulfation was measured by autoradiography (**A**) and quantitated by densitometry (**B**). Total sulfation was determined by precipitation of the remaining lysate. Each bar (mean with SD) represents the average of three independent experiments. The asterisks (p < 0.01) represent significance between % sulfated ricin control and sulfated ricin plus mAb treatment, as determined using an unpaired *t*-test with a 95% confidence interval. While there were slight differences in total sulfation across the different treatment groups (100–115%), none of the differences observed were statistically significant. After the sulfation assay, the membrane was subjected to Western blot analysis with an anti-RTA antibody (**C**) and then quantitated by densitometry (**D**). The densitometry signals in presence of mAbs were normalized to the signal for RS1 alone, which was set to 100%.

To better quantitate the effects of R70 and SyH7 on ricin toxin retrograde trafficking, we employed an organelle-specific sulfation assay in which a derivative of RTA known as RS1 (~30 kDa) becomes modified upon entry into the TGN by resident tyrosylprotein sulfotransferases^17^. RS1 holotoxin was mixed with R70 or SyH7 and then applied to HeLa cells for two hours, after which the cells were washed with lactose to remove surface-associated ricin and then lysed to measure the degree of RS1 sulfation by autoradiography. Sulfation of RS1 was significantly impaired by R70 (>85% reduction) and SyH7 (>60% reduction), but not the non-neutralizing mAb, FGA12 (Fig. 3A,B). The same effect on RS1 sulfation was observed when the experiments were repeated in Vero cells, although SyH7’s effects on RS1 trafficking were slightly more severe (>85%) than was observed in HeLa cells (Supplementary Fig. 3B). These results are consistent with R70 and SyH7 each having an effect on ricin toxin retrograde transport

R70 and SyH7 recognize so-called epitope clusters 1 and 2 on the surface of RTA^9^. We wished to examine what effect other toxin-neutralizing, RTA-specific mAbs, notably IB2 and GD12, which are directed against epitope clusters 3 and 4, have on ricin retrograde transport. IB2 tentatively recognizes a discontinuous epitope at the interface of RTA and RTB, while GD12 recognizes a linear epitope spanning residues α-helix E (residues 163–174) (Supplementary Table 1; Supplementary Fig. 1). We also wished to examine the effect of PB10 on ricin retrograde transport; PB10 is an R70-like mAb that is of interest because it has been fully humanized and is being evaluated as a possible therapeutic for ricin intoxication^7^,^18^. For the sake of licensure purposes, it will be critical to be able to document exactly how PB10 actually neutralizes ricin toxin.

We employed the RS1 sulfation assay to evaluate quantitatively the effect that IB2, GD12 and PB10 have on ricin toxin uptake and retrograde transport to the TGN. As noted above, RS1 holotoxin was mixed with each individual mAb and then applied to HeLa cells that had been grown in the presence of ^35^SO_4_^2−^. We found that IB2, GD12 and PB10 each impaired RS1 sulfation by >80% (Fig. 3A,B). Finally, Western blot analysis of total cell lysates indicated that R70 and SyH7, as well as IB2, GD12 and PB10 actually enhanced (~2-fold) the amount of ricin that was associated with HeLa cells after a 2 hr incubation period, as compared to ricin treated control cells (Fig. 3C,D). Although these differences were not statistically significant (*i.e.*, p > 0.05, as compared to ricin-treated control cells) they do suggest that the mAbs influence the amount of ricin retained on the cell surface or that is internalized by endocytosis. This issue will be discussed in more detail later in the manuscript.

### R70- and SyH7-ricin complexes accumulate in late endosomes and lysosomes

**Figure 4:**
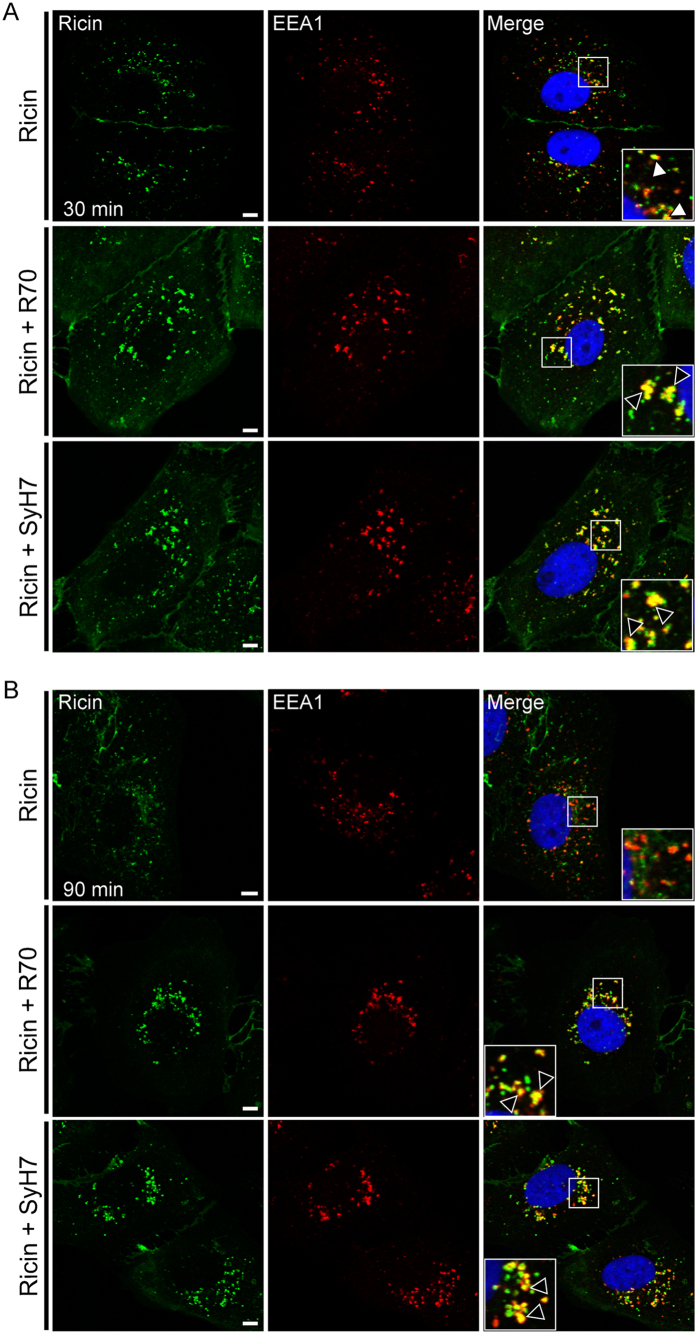
R70 and SyH7 delay ricin egress from EEA-1 vesicles. Vero cells, grown on glass coverslips, were cooled to 4 °C and incubated for 30 min with ricin-FITC. The cells were then washed, treated (or not) with R70 or SyH7 for an additional 30 min at 4 °C and then shifted to 37 °C. At the indicated time points (**A**) 30 min and (**B**) 90 min the cells were fixed, immunolabeled with EEA1 to localize the EEs and imaged by confocal microscopy. Representative images indicating colocalization between ricin (green) and EEA1 (red) staining in the absence of mAb (top panels), or presence of R70 (middle panels), and SyH7 (bottom panels) at 30 min following internalization, but only in the presence of mAbs (bottom and middle panels) at 90 min post internalization (arrowheads). Images are representative of at least 4 independent experiments. Scale bar, 5 μm.

**Figure 5:**
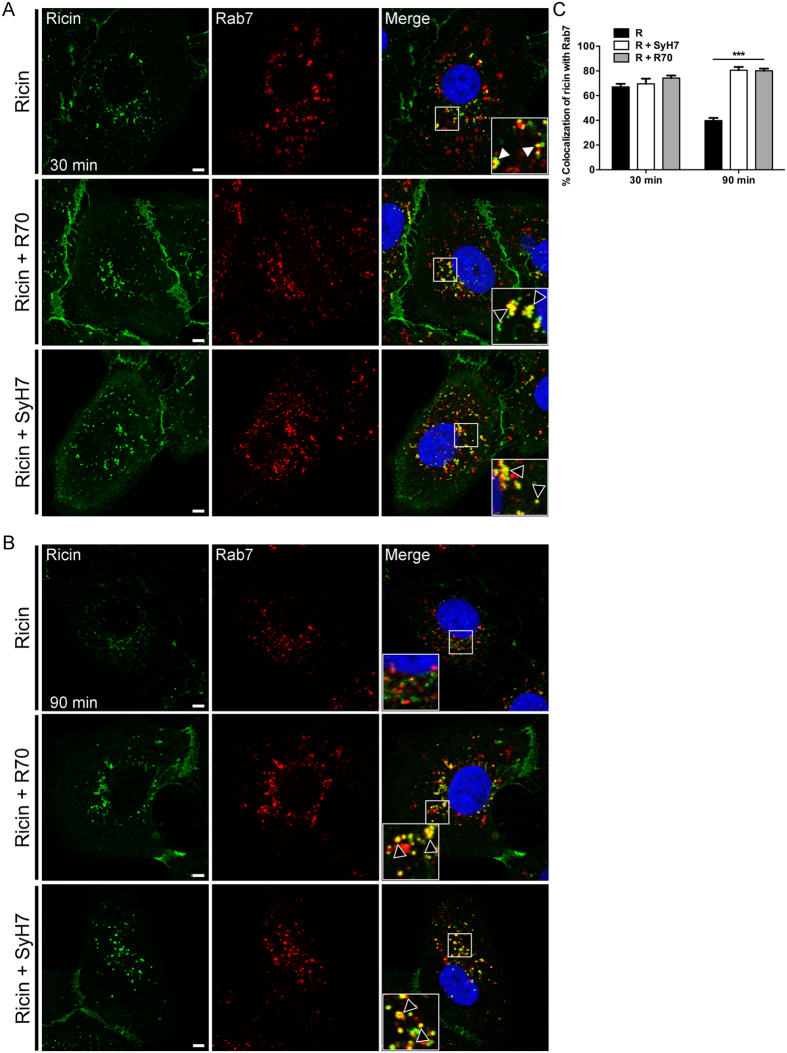
R70 and SyH7 delay ricin egress from Rab-7 vesicles. Vero cells treated at 4 °C with ricin-FITC (top panels), ricin-FITC and R70 (middle panels), or ricin-FITC and SyH7 (bottom panels), as described in the Materials and Methods, were shifted to 37 °C for (**A**) 30 min, or (**B**) 90 min before being fixed and stained for Rab7 (red). Insets (right column) indicate minimal visual colocalization between ricin and Rab7 in the absence of R70 and SyH7, but notable colocalization in the presence of R70 and SyH7 (arrowheads) at both time points. Scale bars, 5 μm. Images are representative of at least three independent experiments. (**C**) The frequency of ricin colocalization with Rab7 at the 30 and 90 min time points were quantitated with ImageJ, as described in the Materials and Methods. Each bar represents the average of approximately 20 cells (with SEM) of 3 individual experiments. ***p < 0.001, determined using a one-way ANOVA with Tukey’s post-test.

To better define the fate of mAb-ricin complexes within cells, we subject Vero cells to staining with EEA-1, Rab-7 and Rab-11, which are markers of early (EE), late (LE), and recycling (RE) endosomes, respectively. In toxin-only treated cells, ricin was observed in EEA-1^+^ vesicles and Rab-7^+^ vesicles at the 30 and 90 min time points (Figs 4 and 5). Similarly, analysis of cells treated with ricin and R70 or SyH7 revealed that toxin was present in EEA-1^+^ vesicles and Rab-7^+^ vesicles at the 30 and 90 min time points (Figs. 4 and 5). However, when treated with R70 or SyH7, ricin’s co-localization with Rab-7^+^ vesicles at 90 min was significantly increased (Figs. 4 and 5), suggesting that the possibility that antibody-toxin complexes are retained in LE. Neither R70 nor SyH7 affected ricin’s association with RE (Supplementary Fig. 4), indicating that expulsion of toxin-antibody complexes via RE is not enhanced.

**Figure 6:**
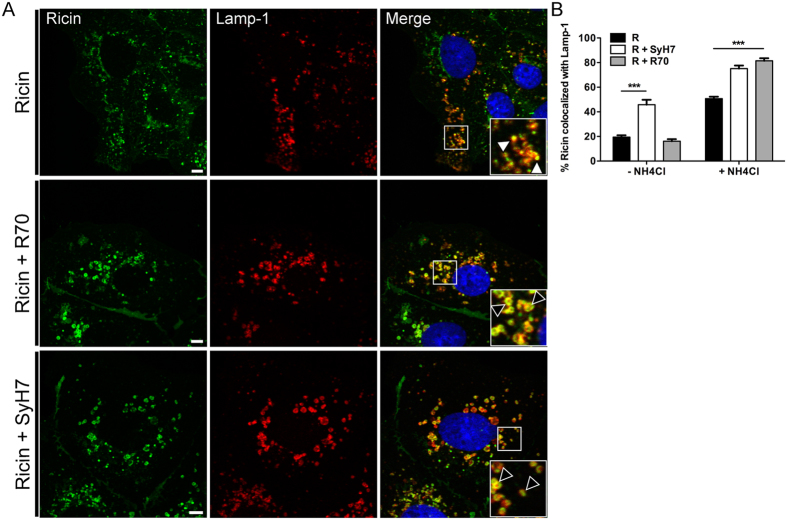
R70 and SyH7 promote ricin accumulation in Lamp-1^+^ vesicles. Vero cells were treated at 4 °C with R70 or SyH7 and ricin-FITC, in the absence or presence of NH_4_Cl to prevent lysosome acidification, and then shifted to 37 °C for 4.5 hr. (**A**) Vero cells were then fixed and stained with Alexa Fluor-647 labeled Lamp-1 antibodies. NH_4_Cl treatment resulted in the accumulation of ricin-R70 or ricin-SyH7 containing vesicles that were positive for Lamp-1 (arrowheads within insets, bottom right panels). Scale bar, 5 μm. (**B**) The frequency of ricin (in the absence or presence of R70 or SyH7) colocalization with Lamp-1 in cells treated or not with NH_4_Cl. Each bar represents the average of 25–30 cells (with SEM) per time point. ***p < 0.001, determined using a one-way ANOVA with Tukey’s post-test.

**Figure 7:**
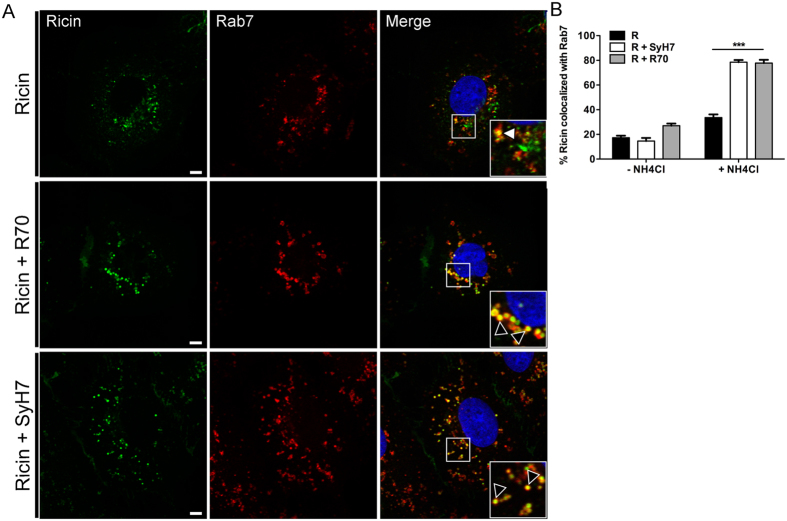
R70 and SyH7 promote the accumulation of ricin in Rab7 ^+^ vesicles when endosome acidification is inhibited. Vero cells were treated at 4 °C with R70 or SyH7 and ricin-FITC, in the absence or presence of NH_4_Cl to prevent lysosome acidification, and then shifted to 37 °C for 4.5 hr. Vero cells were then fixed and immunolabeled with Rab7 antibodies. (**A**) NH_4_Cl treatment resulted in the accumulation of ricin-mAb containing vesicles that were positive for Rab7 (arrowheads within insets, middle and bottom right panels). Scale bar, 5 μm. (**B**) The frequency of ricin (in the presence of mAbs) colocalization with Rab7 in cells treated or not with NH_4_Cl. Each bar represents the average of 25–30 cells (with SEM) per time point. ***p < 0.001, determined using a one-way ANOVA with Tukey’s post-test.

Based on these observations we speculated that R70 and SyH7 might promote the trafficking of ricin to lysosomes for degradation. To address this experimentally, Vero cells were treated with 10 mM NH_4_Cl, an inhibitor of endosome-lysosome acidification, prior to being challenged with R70- and SyH7-toxin complexes. Cells were fixed 4.5 hr later and visualized by confocal microscopy. In the absence of NH_4_Cl, ricin-mAb complexes were detected within small diffusely localized vesicles that were only moderately fluorescent, possibly indicative of ricin-mAb complexes undergoing lysosome-mediated degradation. In contrast, NH_4_Cl treatment resulted in the accumulation of ricin-mAb complexes in large, fluorescently bright vesicles (Supplementary Fig. 5). Immunolabeling confirmed that the vesicles were positive for Lamp-1, a well-recognized marker of lysosomes (Fig. 6A). The large size of the vesicles was likely due to swelling of the lysosomes caused by NH_4_Cl treatment and not ricin accumulation, because lysosomotropic compounds like NH_4_C are known to promote lysosome enlargement and swelling^14^,^19^,^20^. In the presence of NH_4_Cl, ricin-mAb complexes were more frequently associated with LAMP-1^+^ and Rab7^+^ vesicles than was ricin alone (Figs. 6B and 7), supporting a model in which ricin-mAb complexes are preferentially shunted to lysosomes for degradation.

### Interference of ricin toxin retrograde transport by vaccine-induced immune sera

**Figure 8:**
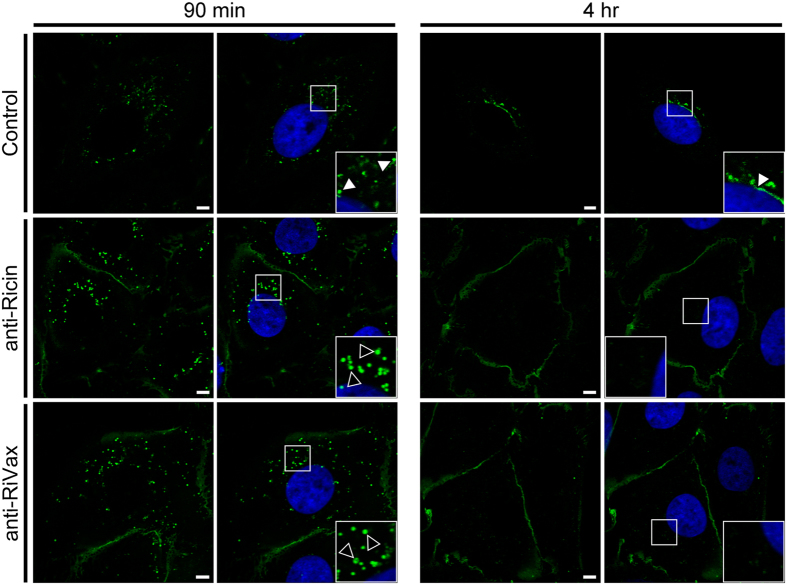
Polyclonal RTA-specific Abs interfere with ricin trafficking to perinuclear compartment. Vero cells, grown on glass coverslips, were cooled to 4 °C and incubated for 30 min with ricin-FITC. The cells were then washed, treated with naïve (control), anti-ricin and anti-RiVax polyclonal mouse sera for an additional 30 min at 4 °C and then shifted to 37 °C. At the indicated time points (90 min and 4 hr) the cells were fixed and imaged by confocal microscopy. Insets in the right and left hand columns highlight the subcellular localization of ricin (+naïve sera, white arrowheads) and ricin-sera complexes (black arrowheads). Scale bar, 5 μm.

RiVax is candidate ricin toxin subunit vaccine consistning of a full-length, non-toxic recombinant derivative of RTA^21^. RiVax vaccination of mice elicits high levels of circulating RTA-specific antibodies that protect mice against lethal toxin challenge^22^. However, the mechanism(s) by which anti-ricin and anti-RiVax polyclonal antibodies neutralize ricin has not been investigated. To address the possibility that polyclonal anti-RTA antisera impact ricin retrograde trafficking, ricin-FITC was mixed with antiserum from a mouse treated three times with sub-lethal doses of ricin (unpublished results) or antiserum from a mouse vaccinated with RiVax^23^. The antibody/ricin-FITC mixtures were applied to Vero cells at 4 °C and then shifted to 37 °C to permit internalization. Examination of cells at 90 min and 4 hr time points revealed that each antiserum impacted toxin trafficking profiles (Fig. 8). At 90 min, ricin that had been treated with antisera was present in vesicles that were larger and brighter than ricin without antibody treatment. By 4 hr, there was little to no detectable intracellular ricin in cells that had been treated with ricin or RiVax antisera, suggesting the toxin-antibody complexes had been cleared from the cell through lysosomal degradation.

## Discussion

Ricin toxin’s enzymatic subunit, RTA, is at the center of efforts to develop a safe and effective ricin toxin subunit vaccine for use by military personnel, laboratory research staff, and emergency first responders^5^,^24^,^25^. Chimeric and fully humanized RTA-specific mAbs are also being pursued as possible therapeutics to treat individuals suffering ricin intoxication^5^,^18^. Despite the increased interest in ricin’s enzymatic subunit, it remains largely unclear how RTA-specific antibodies neutralize ricin toxin. Two reports published in 2013 suggested that RTA-specific mAbs likely neutralize ricin toxin intracellularly, rather than extracellularly^12^,^15^. We demonstrated by confocal microscopy that ricin-IB2 complexes are internalized into Vero cells, although the effects of the antibody on retrograde trafficking were not investigated^15^. Song and colleagues used live cell imaging to track the kinetics of ricin uptake into HeLa cells in the presence of the neutralizing mAb RAC18^12^. They observed that RAC18 was internalized into host cells with ricin and delayed the toxin trafficking to the ER.

In the current study we have now refined those two previous reports and demonstrate that two well-characterized mAbs, R70 and SyH7, as well as three additional toxin-neutralizing mAbs, GD12, IB2 and PB10, interfere with ricin retrograde transport from the plasma membrane to the TGN, presumably by shunting ricin to lysosomes for degradation. The five mAbs recognize at least four spatially distinct epitopic regions on the surface of RTA: R70 and PB0 bind linear epitopes on the “front side” of RTA focused around α-helix E (residues 97–108), while SyH7 recognizes a linear epitope on the “back side” of RTA around α-helix F (187–198). GD12 binds a linear epitope spanning residues 163–174 that corresponds to α-helix E^8^. The exact epitope recognized by IB2 is not known, but is postulated to span the interface between RTA and RTB^15^.

Although we demonstrated that five different RTA-specific mAbs can interfere with ricin toxin retrograde transport, we have not elucidated the mechanism(s) by which this occurs. It is possible that physical association of a single mAb (1:1 antibody:toxin ratio) with one or two molecules (1:2 antibody:toxin ratio) of ricin may be sufficient to derail toxin trafficking. It was shown many years ago that a monovalent ricin-HRP conjugate (30 kDa) does not alter retrograde transport, but polyvalent ricin-HRP or colloidal gold conjugates did, suggesting that valency and/or size of the conjugate affects how the cell sorts the toxin^16^. Unfortunately, we now know that our choice of FGA12 as a non-neutralizing negative control mAb in this study does not help resolve the issue of whether or not the association of a mAb with ricin is sufficient to interfere with trafficking. When first described, FGA12 was characterized as being able to bind RTA by ELISA but devoid of any detectable toxin-neutralizing activity *in vitro* or *in vivo*^10^. Pepscan analysis revealed that FGA12 recognizes a linear epitope in RTA’s N-terminus (residues 37–48). Based on these attributes, we rationalized that FGA12 would serve as an ideal control for the present study (i.e., binds ricin but fails to neutralize). However, in the past year we have discovered that FGA12 recognizes a “cryptic” epitope on RTA that is exposed when RTA or ricin is adsorbed to polystyrene microtiter ELISA plates, but that is sequestered on RTA or ricin in solution (J. O’Hara, D. Vance, and N. Mantis, manuscript in preparation). As such, FGA12 fails to recognize ricin in solution. Two other non-neutralizing mAbs, SB1 and BD12 share the same characteristics (e.g., recognize cryptic epitopes) even though all three mAbs recognize different epitopes^10^. The upshot of this is that we cannot formally exclude the possibility that the association any mAb to the surface of RTA (irrespective of epitope) is sufficient to affect ricin trafficking and thereby neutralizing the toxin.

Nonetheless, as shown in Fig. 3, it is interesting that all five neutralizing mAbs tested in this study (i.e., R70, PB10, SyH7, GD12, and IB2) increased the amount of ricin that was associated with host cells after a two hour incubation period, followed by a lactose wash to remove surface bound toxin. Although these differences were not statistically significant (as compared to ricin alone), the numbers do suggest the mAbs influence the dynamics of ricin on the cell surface or within vesicular compartment(s). Song and colleagues suggested that the neutralizing mAb RAC18 promotes toxin accumulation on the cell surface and delays toxin uptake^12^. Our results are consistent with R70, PB10, SyH7, GD12, and IB2 having similar effects on ricin. As noted above, we are particularly interested in the possibility that neutralizing mAbs influence the valency of ricin at the cell membrane and, thus, alter receptor crosslinking and endocytosis^26^. Indeed, we have demonstrated that recombinant, bispecific camelid antibodies are particularly potent at neutralizing ricin *in vitro* and *in vivo*^27^. That said, crosslinking alone cannot explain toxin-neutralizing activity, as monovalent Fab fragments of R70, for example, are able to neutralize ricin as effectively as IgG^28^.

The results from the current study will have implications for the development of countermeasures against ricin toxin. As noted in the Results section, PB10 is of particular interest to us because it is being evaluated as a possible therapeutic for ricin intoxication. We previous described chimeric version of PB10 in which the murine V_H_ and V_L_ domains were grafted onto a human IgG1 framework^18^. The chimeric version of PB10 was expressed in a *Nicotiana benthamiana*-based rapid antibody-manufacturing platform (RAMP) that provides the potential for extremely fast and high-yield monoclonal antibody (MAb) production. Chimeric PB10 has potent ricin toin-neutralizing activity *in vitro* and *in vivo*, including the ability to rescue mice from the effects of ricin when administered up to 3 to 4 hr after toxin challenge. We have now produced a fully humanized version of PB10 in which the murine V_H_ and V_L_ framework regions have been mutated to conform with human consensus sequences (M. Pauly, L. Zeitlin, K. Whaley, E. Sully, G. Van Slyke, and N. Mantis, manuscript in preparation). The fully humanized version of PB10 completely neutralizes ricin *in vitro* and in mouse models of systemic and mucosal ricin challenge. We also demonstrate that anti-RiVax antiserum interferes with ricin retrograde transport *in vitro*, suggesting that this activity may be associated with vaccine-induced immunity to ricin. Future studies will be aimed at resolving exactly how mAbs and polyclonal antibodies affect trafficking of ricin within the endosomal system.

## Methods

### Chemicals, biological reagents, supplies and cell lines

Ricin toxin (*Ricinus communis* agglutinin II) and ricin-FITC were purchased from Vector Laboratories (Burlingame, CA). Tween-20, Triton X-100, and Hoechst 33342 were purchased from Sigma-Aldrich (St. Louis, MO). Commercial secondary and primary Abs are described in Supplementary Table 2. Glass coverslips (22 × 22 mm square, 1.5 mm) were purchased from Corning-Fischer Scientific (Suwanee, GA). Tissue culture-treated dishes (35 mm x 15 mm) were purchased from CELLTREAT Scientific Products (Shirley, MA). Cytofix/Cytoperm™ Fixation/Permeabilization Solution was purchased from BD Biosciences (San Jose, California). Ammonium chloride was purchased from Sigma-Aldrich. HeLa and Vero cells were purchased from the American Type Culture Collection (Manassas, VA). Unless otherwise noted, all cell lines were maintained in a humidified incubator at 37 °C with a 5% CO_2_ atmosphere.

### Ricin mAbs and RiVax antiserum from mice

Murine mAbs have been described previously^10^ and were purified from hybdridoma supernatants using ion-exchange (IEX) and protein G chromatography under endotoxin-free conditions. The R70 (also known as UNIVAX70/38) hybridoma was originally obtained from American Type Culture Collection (Manassas, VA). Archived RiVax antiserum was available from a previous study in which female BALB/c mice had been immunized with RiVax-adsorbed to Alhydrogel by the subcutaneous route^23^. Those animal experiments were approved by and performed in strict accordance with the Wadsworth Center’s Institutional Animal Care and Use Committee (IACUC) under protocol 13–384. The Wadsworth Center complies with the Public Health Service Policy on Humane Care and Use of Laboratory Animals. Moreover, the Wadsworth Center is fully accredited by the Association for Assessment and Accreditation of Laboratory Animal Care (AAALAC). Obtaining this voluntary accreditation status reflects that Wadsworth Center’s Animal Care and Use Program meets all of the standards required by law, and goes beyond the standards as it strives to achieve excellence in animal care and use.

### Ricin-specific sulfation assays

Ricin-sulf-1 (RS1), ricin with a modified ricin A-subunit containing a tyrosine sulfation site, was produced and purified as described previously^17^,^29^. HeLa and Vero cells were washed with sulfate-free HEPES-buffered medium supplemented with 2 mM L-glutamine, followed by incubation with 0.2 mCi/ml Na_2_^35^SO_4_ (Hartmann Analytic, Braunschweig, Germany) in sulfate-free HEPES-buffered medium for 2.5 hr at 37 °C. The radioelement 35S used in this study was handled according to the national guidelines given by the Norwegian Radiation Protection Authority. The work was performed in specific designated areas, using proper protective gear.

RS1 was pre-incubated with the indicated anti-RTA mAbs for 30 min at room temperature, before the mixture was applied to cells and incubated for 2 hr at 37 °C. The cells were then washed (2 × 5 min) with 0.1 M lactose in HEPES-buffered medium, and once in cold PBS on ice before the addition of 400 μl lysis buffer (0.1 M NaCl, 10 mM Na_2_HPO_4_, 1 mM EDTA, 1% Triton X-100, 60 mM octyl glycopyranoside) supplemented with complete protease inhibitors (Roche Diagnostics, Mannheim, Germany). The lysate was cleared by centrifugation (8000 rpm, 10 min, 4 °C) and 300 μl of the supernatant was mixed with 1 ml 5% trichloroacetic acid (TCA) followed by centrifugation at 14,000 rpm (10 min, 4 °C). The resulting pellet was washed once in ice-cold PBS, dissolved in 2x sample buffer and subjected to SDS-PAGE under reducing conditions, followed by blotting onto a PVDF membrane (Immobilon-P, Millipore, Billerica, MA, USA). A ^14^C-methylated protein molecular weight standard (^14^C Standard, PerkinElmer, Waltham, MA) was subject to SDS-PAGE alongside the cell lysates. The bands were detected by autoradiography using a PharosFX scanner and quantified using Quantity One® 1-D Analysis Software (BioRad Laboratories Inc, Hercules, CA, USA). The total amount of sulfated proteins was determined by TCA-precipitation of the remaining lysates.

For the purpose of quantification of ricin internalization after the sulfation assay, the resulting PVDF membrane was re-wet in PBS-T (PBS with 0.01% Tween-20) and then probed overnight at 4 °C with polyclonal anti-RTA antibody (Abcam, Cambridge, MA) in 5% BSA in PBS-T. The membrane was then repeatedly washed with PBS-T and probed with HRP-conjugated secondary antibody (Jackson Immunoresearch) that had been diluted in 1% BSA in PBS-T. The membrane were developed using ECL Western blotting detection reagent (GE Healthcare, Buckinghamshire, UK) and quantified using Quantity One® 1-D Analysis Software (BioRad, Oslo, Norway). The densitometry signals in presence of antibodies were normalized to the signal for RS1 alone, which was set to 100%.

### Analysis of ricin endocytosis by confocal fluorescence microscopy

As described previously^14^, Vero cells were seeded onto sterile glass coverslips, cooled to 4 °C for 30 min before the addition of ricin-FITC (10 μg/coverslip) and then incubated for an additional 30 min. The coverslips were rinsed with 10% FBS in DMEM (at 4 °C) then stained with anti-ricin mAbs (5 μg/sample) or sera (5 μg/ sample). The coverslips were incubated at 4 °C for 30 min before being transferred to tissue culture dishes containing pre-warmed medium and incubated at 37 °C for specific time points (30–320 min) prior to fixation with 4% PFA, and permabilization with 0.5% Triton X-100. Cells were washed and blocked with PBS-Tween (0.5%) supplemented with superblock solution (5% normal goat serum and 5% BSA) for staining with anti-mouse-secondary, anti-EEA1 or anti-Golgin97 antibodies, or superblock plus solution (5% normal goat serum, 5% BSA and 5% CARNATION® Instant Nonfat Dry Milk) for anti-Rab7 and anti-Rab11 staining. Incubation with Rab7 and Rab11 Abs was done overnight at room temperature, while EEA1 and Golgin97 Abs incubations were done for 1 hr at 37 °C. Anti-Lamp-1 antibody staining was performed as per the instructions in the BD Cytofix/Cytoperm™ Fixation/Permeabilization Solution Kit. Hoechst (1 μg/ml) was used to stain cell nuclei. For experiments involving NH_4_Cl, cells were pre-treated with media containing 10 mM NH_4_Cl for 20 min at 37 °C, prior to chilling and binding of ricin and anti-ricin mAbs. Ten mM NH_4_Cl was maintained in media throughout the remainder of experiments.

Cells were imaged as described previously^14^ using a Leica TCS SP5 AOBS (acousto-optical beam splitter) confocal microscope with multi-photon laser and a 63x objective (1.4 NA) in a sequential manner (Leica Microsystems Inc. Buffalo Grove, IL). Z-stacks were collected for (20–35) steps 0.13 μm apart. The frequency of ricin-FITC colocalization with Rab7, Lamp-1 or Golgin97 was determined using Manders’ coefficients within the Just Another Colocalisation Plugin (JACoP) for ImageJ^30^. Ricin-FITC positive vesicles were selected using ImageJ’s freehand selection option and percent colocalization was determined by measuring the selected fraction of ricin-FITC that overlaps with Rab7 or Lamp-1 vesicles or Golgin97.

### Statistical analysis and software

Statistical analysis was carried out with GraphPad Prism 5 (GraphPad Software, San Diego, CA). Microscopy image processing and analysis were done using ImageJ 1.46j (public domain) and Adobe Photoshop CS4 (Adobe Systems Inc., San Jose, CA).

## Additional Information

**How to cite this article**: Yermakova, A. *et al*. Neutralizing Monoclonal Antibodies against Disparate Epitopes on Ricin Toxin’s Enzymatic Subunit Interfere with Intracellular Toxin Transport. *Sci. Rep.* **6**, 22721; doi: 10.1038/srep22721 (2016).

## Supplementary Material

Supplementary Information
