# Supplementary material for: Neutralizing Monoclonal Antibodies against Disparate Epitopes on Ricin Toxin’s Enzymatic Subunit Interfere with Intracellular Toxin Transport
Source: Sci Rep. 2016 Mar 7;6:22721. doi: 10.1038/srep22721 (PMC4779987; doi:10.1038/srep22721)

Supplementary Information

Manuscript: Neutralizing Monoclonal Antibodies against Disparate Epitopes on the Ricin Toxin's Enzymatic Subunit Interfere with Intracellular Toxin Transport

Authors and Affiliations: Anastasiya Yermakova[1,2], Tove Irene Klokk[3], Joanne M. O'Hara[1,2], Richard Cole[2,5], Kirsten Sandvig[3,4], and Nicholas J. Mantis[1,2*]
[1]Division of Infectious Disease, Wadsworth Center, New York State Department of Health, Albany, NY 12208; [2]Department of Biomedical Sciences, School of Public Health, University at Albany, Albany, NY 12201; [3]Department of Molecular Cell Biology, Centre for Cancer Biomedicine, Institute for Cancer Research, The Norwegian Radium Hospital, Oslo University Hospital, Montebello, Oslo, Norway; [4]Department of Biosciences, University of Oslo, Oslo, Norway; [5]Division of Translational Medicine, Wadsworth Center, New York State Department of Health, Albany, NY 12201;

Supplementary Tables

Table S1. RTA-specific mAbs used in this study.

mAb	subclass	Epitope[a]	Epitope Cluster	neutralizing[b]
R70	IgG1	97-108	I	+
PB10	IgG2b	97-108	I	+
SyH7	IgG1	187-198	II	+
GD12	IgG1	163-174	III	+
IB2	IgG1	n.d.	IV	+
FGA12	IgG1	37-48	n.d.	-

Table S2. Primary and secondary antibodies used in in this study.

Target	Dilution	Species[a]	Conjugate	Vendor[b]
Rab7	1:30	R	Unlabeled (UL)	Cell Signaling Technology
Rab11	1:50	R	UL	Cell Signaling Technology
EEA1	1:100	R	UL	abcam
Golgin97	1:40	R	UL	abcam
Lamp-1	1:20	M	Alexa Fluor® 647	BioLegend
anti-mouse IgG (H+L)	1:50	G	PerCP	Jackson ImmunoResearch
anti-mouse IgG (H+L)	1:300	G	DyLight® 650	Leinco Technologies
anti-mouse IgG (H+L)	1:200	R	DyLight® 549	Jackson ImmunoResearch
anti-rabbit IgG (H+L)	1:200	G	DyLight® 649	Jackson ImmunoResearch
anti-rabbit	1:200	G	Alexa Fluor® 633	Life Technologies
anti-mouse	1:200	G	Alexa Fluor® 546	Life Technologies

[a]Source species; R, rabbit; M, mouse; G, goat. [b]Cell Signaling Technology (Beverly, MA); abcam (Cambridge, MA); BioLegend (San Diego, CA); Jackson ImmunoResearch (Bar Harbor, ME); Leinco Technologies (Fenton, MO); Life Technologies (Carlsbad, CA).

Supplementary Figure Legends

Figure S1: B cell epitopes on the surface of RTA. Surface depictions of the front, side and back of ricin toxin (PDB: 2AAI). Ricin binding subunit (RTB) is shown in grey. RTA is shown in gold, the active site in black and specific epitopes indicated by as follows: PB10 (red); SyH7 (salmon); FGA12 (violet). Modeling was done using PyMOL(PyMOL Molecular Graphics System, Version 1.7.4 Schrödinger, LLC).

Figure S2: SyH7 internalized in complex with ricin and reduces toxin accumulation in the perinuclear compartment. Vero cells, grown on glass coverslips, were cooled to 4°C and incubated for 30 min with ricin-FITC. The cells were then washed, treated (or not) with SyH7 for an additional 30 min at 4°C and then shifted to 37°C. At the indicated time points (30 min, 90 min and 4 h) the cells were fixed, stained with **DyLight® 549 labeled** secondary Ab and imaged by confocal microscopy. Insets in the right and left hand columns highlight the subcellular localization of ricin (white arrowheads) and ricin-SyH7 complexes (black arrowheads). Images are representative of at least 5 independent experiments. Scale bar, 5 µm.

Figure S3: R70 and SyH7, but not FGA12 interfere with the trafficking of ricin in Vero and HeLa cells. (Panel A) Vero cells, grown on glass coverslips, were cooled to 4°C and incubated for 30 min with ricin-FITC. The cells were then washed, treated (or not) with **FGA12** for an additional 30 min at 4°C and then shifted to 37°C. At the indicated time points (30 min, 90 min and 4 hr) the cells were fixed, stained with **DyLight® 549 labeled** secondary Ab and imaged by confocal microscopy. Insets in the right and left hand columns highlight the subcellular localization of ricin. Images are representative of at least 2 independent experiments. Scale bar, 5 µm. (**Panel B**) Cells were incubated with $Na_2{}^{35}SO_4$ prior to the addition of RS1 in the absence or presence of the indicated mAbs. Two hr later the cells were washed with buffer containing lactose to remove any residual surface bound ricin and then lysed. Precipitated proteins from lysates were subjected to SDS-PAGE and transferred to a PVDF membrane. Specific RTA sulfation was measured by autoradiography and quantitated by densitometry. Total sulfation was determined by precipitation of the remaining lysate. Each bar (mean with SD) represents the average of two independent experiments.

Figure S4: R70-ricin and SyH7-ricin containing vesicles do not fuse with $Rab11^+$ vesicles. Vero cells, grown on glass coverslips, were cooled to 4°C and incubated for 30 min with ricin-

FITC. The cells were then washed, treated (or not) with R70 or SyH7 for an additional 30 min at 4°C and then shifted to 37°C. At 90 min, the cells were fixed, immunolabeled with Rab11 to localize the recycling endosomes and imaged by confocal microscopy. Representative images demonstrating the lack of colocalization between ricin (green) and Rab11 (red) staining in the absence (top panel) or presence of R70 (middle panel), or SyH7 (bottom panel), at 90 min following internalization. Images are representative of at least 2 independent experiments. Scale bar, 5 μm.

Figure S5: Ricin in complex with R70 or SyH7 accumulates in large vesicles when endosome acidification is inhibited. Vero cells were treated at 4°C with R70 or SyH7 and ricin-FITC, in the absence or presence of 10 mM NH_4Cl to prevent lysosome acidification, and then shifted to 37°C for 4.5 hr. Vero cells were then fixed and stained with DyLight 650-labeled goat anti-mouse IgG Fabs to detect (**A**) R70 or (**B**) SyH7. NH_4Cl treatment resulted in the accumulation of ricin-R70 and ricin-SyH7 containing vesicles (arrowheads within insets, bottom right panels). Scale bar, 5 μm.

Figure S1

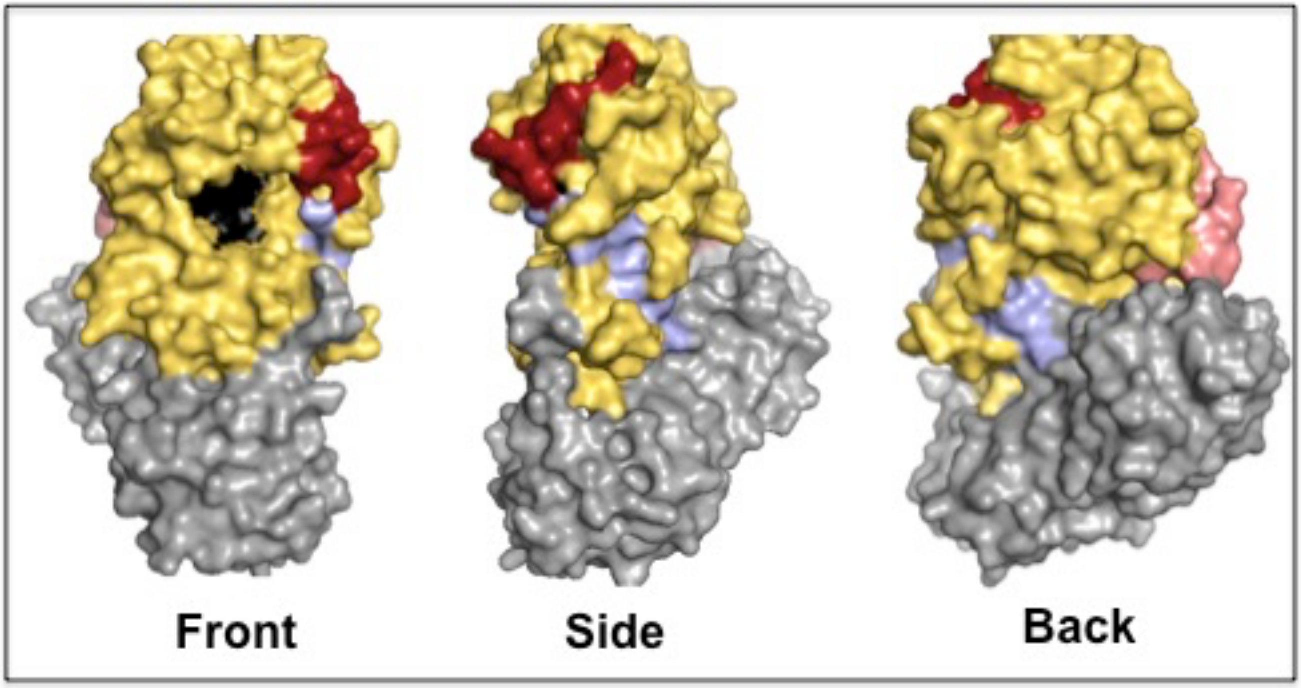

Figure S2

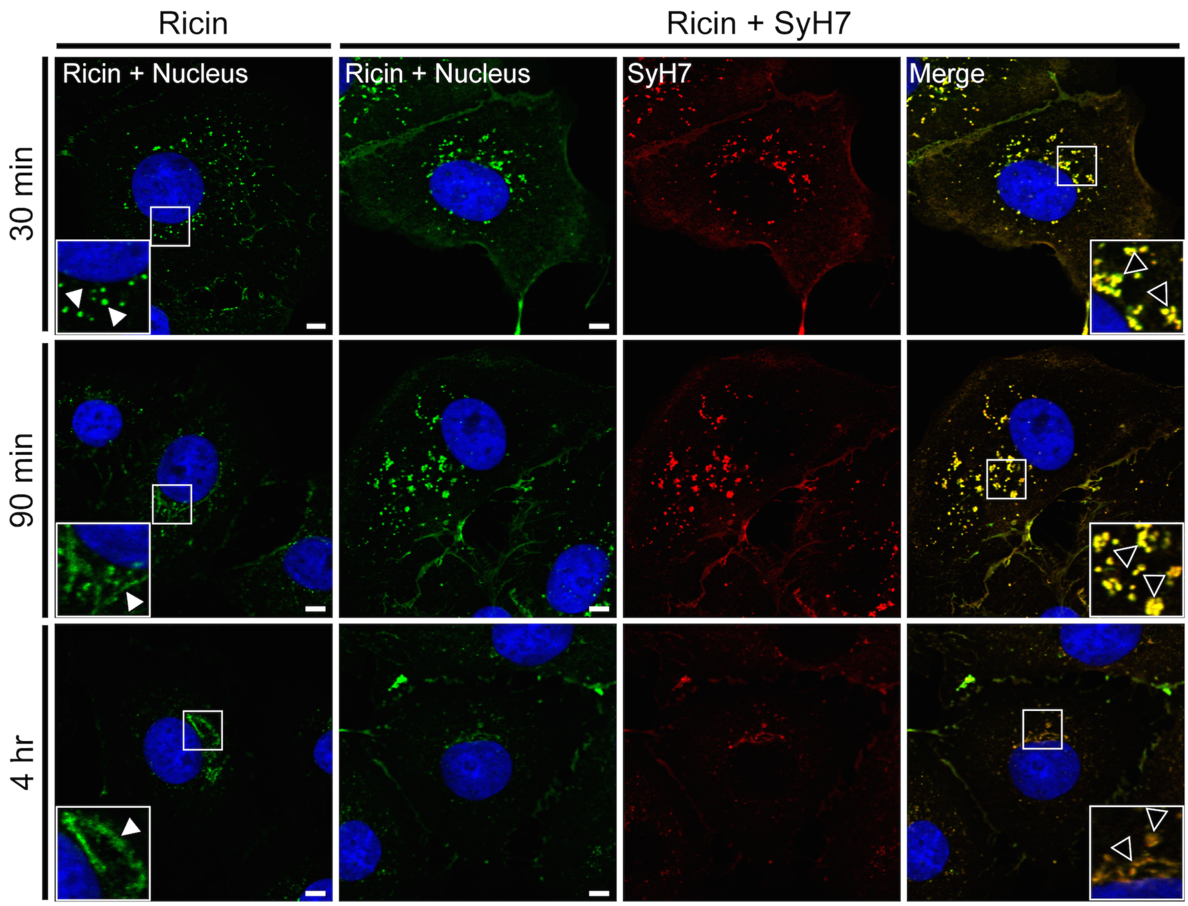

Ricin
Ricin + SyH7
Ricin + Nucleus
Ricin + Nucleus
SyH7
Merge
30 min
90 min
4 hr

Figure S3

A

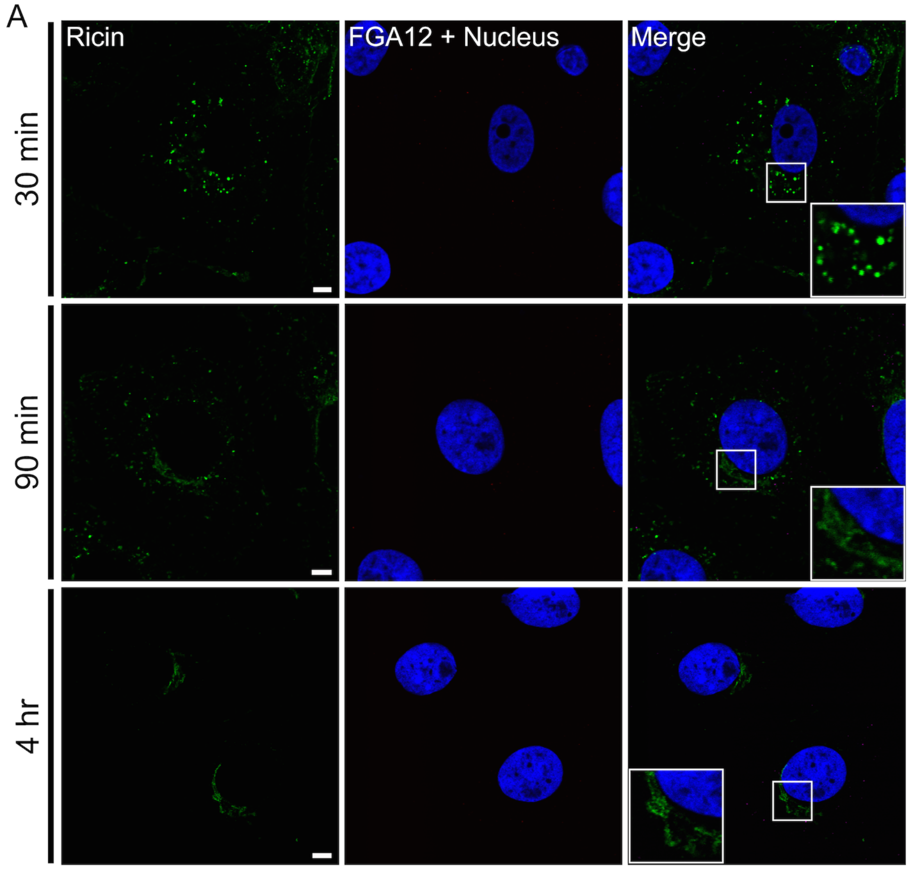

Ricin
FGA12 + Nucleus
Merge
30 min
90 min
4 hr

B

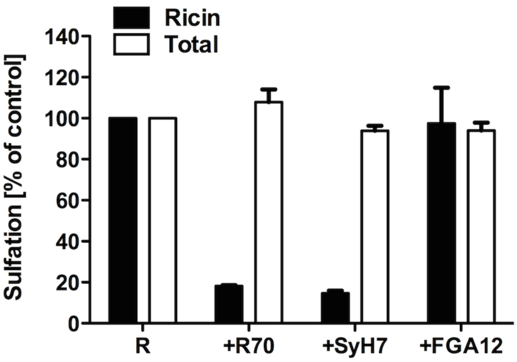

Ricin
Total
Sulfation [% of control]
140
120
100
80
60
40
20
0
R
+R70
+SyH7
+FGA12

Figure S4

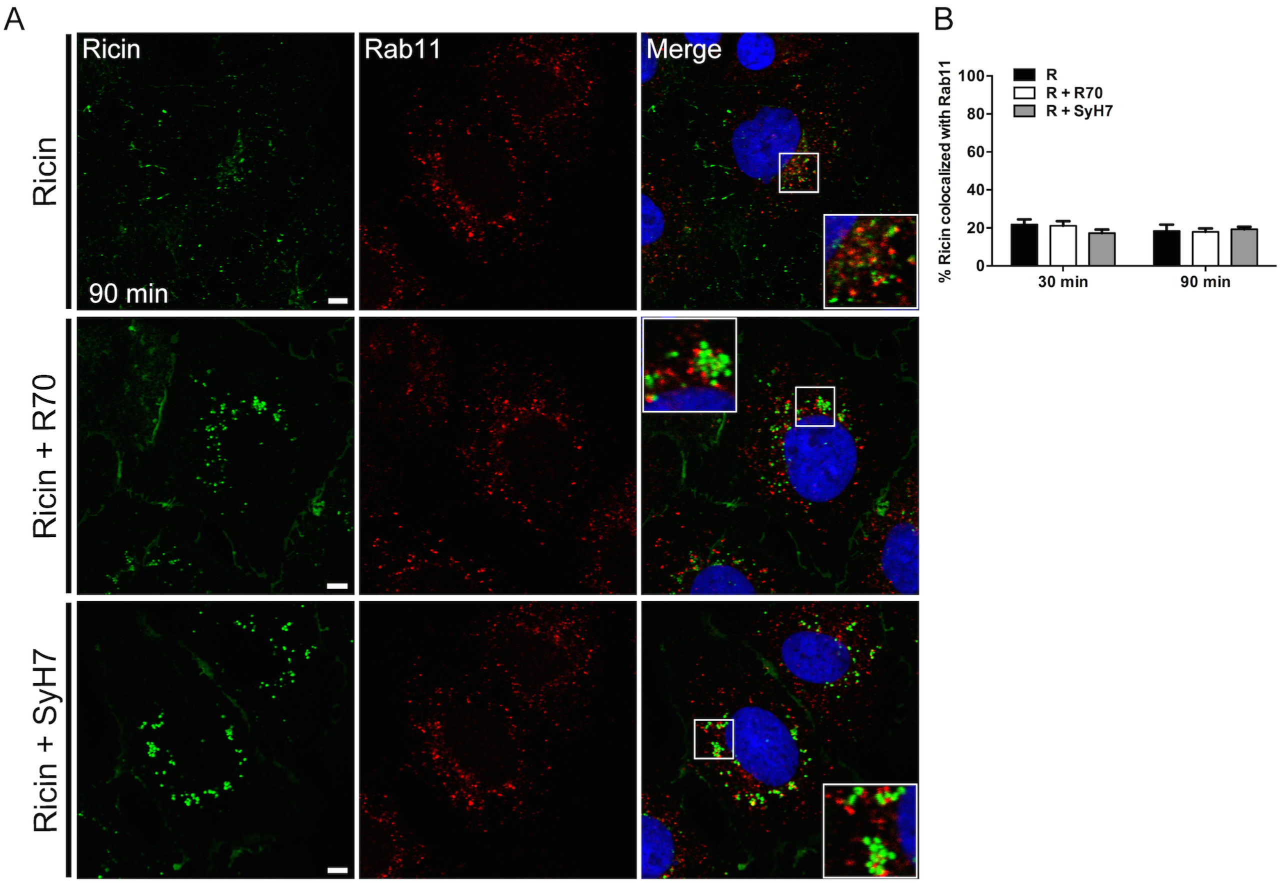
A
Ricin
Rab11
Merge
Ricin
90 min
Ricin + R70
Ricin + SyH7
B
% Ricin colocalized with Rab11
100
80
60
40
20
0
R
R + R70
R + SyH7
30 min
90 min

Figure S5

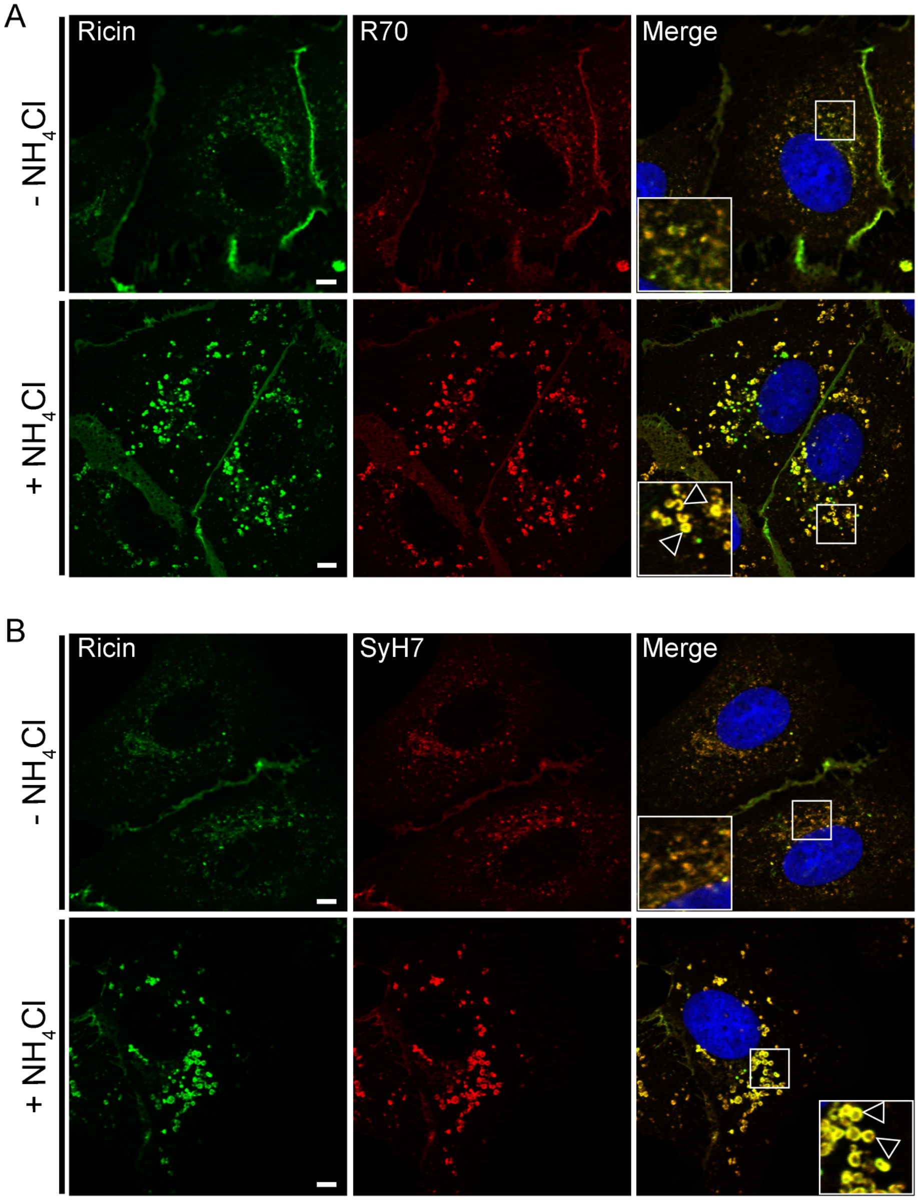

Supplement: Supplementary Information [file srep22721-s1.pdf]
